# Exploring the anti-metastatic potential of sunitinib and novel analogs in colorectal cancer: insights into HIF-1α mediated metastasis

**DOI:** 10.3389/fphar.2025.1520881

**Published:** 2025-02-04

**Authors:** Fatemeh B. Rassouli, Maryam M. Matin, Farzin Hadizadeh, Masoud Nejabat, Hossein Allahverdizadeh, Hamidreza Jamali, Shahin Gharedaghi, Halimeh Hassanzadeh

**Affiliations:** ^1^ Novel Diagnostics and Therapeutics Research Group, Institute of Biotechnology, Ferdowsi University of Mashhad, Mashhad, Iran; ^2^ Department of Biology, Faculty of Science, Ferdowsi University of Mashhad, Mashhad, Iran; ^3^ Biotechnology Research Center, Pharmaceutical Technology Institute, Mashhad University of Medical Sciences, Mashhad, Iran; ^4^ Department of Medicinal Chemistry, School of Pharmacy, Mashhad University of Medical Sciences, Mashhad, Iran; ^5^ Stem Cell and Regenerative Medicine Research Group, Academic Center for Education, Culture and Research (ACECR)-Khorasan Razavi, Mashhad, Iran

**Keywords:** colon cancer, metastasis, sunitinib, novel analogs, hypoxia

## Abstract

**Introduction:**

Colorectal cancer (CRC) is a prevalent malignancy worldwide with high mortality rate. Metastasis, the primary cause of cancer-related deaths, is attributed to various factors including tumor hypoxia. Due to the urgent demand for potent anti-metastatic agents, we aimed to determine the effects of sunitinib and novel analogs on the metastatic behavior of human CRC cells in hypoxic condition for the first time.

**Methods:**

For *in silico* analyses, pathogenic targets of metastatic CRC were identified, PPI network was constructed and KEGG pathway enrichment analysis was conducted. The expression of *HIF1A* was evaluated in seven CRC cell lines, and computational modeling was carried out to define the interaction of sunitinib with HIF-1α. For *in vitro* studies, analogs of sunitinib were synthesized, and cells were assessed for viability, migration, invasion, MMPs activity and gene expression in hypoxic condition.

**Results and Discussion:**

Computational analyses highlighted the importance of HIF-1α as a crucial mediator of metastasis in CRC. Molecular docking and dynamics simulations demonstrated favorable and stable interaction of sunitinib and three novel analogs with HIF-1α PAS-B domain. Volcano plots indicated upregulation of *HIF1A* in LoVo cells compared to six other CRC cell lines. Findings of *in vitro* studies revealed considerable inhibitory effects of sunitinib and analogs on LoVo cell migration and invasion in hypoxic condition. Gelatin zymography and qPCR analysis indicated decreased activity of MMP-2 and MMP-9, along with downregulation of EMT transcription factors in hypoxic condition. Current study reports promising anti-metastatic effects of sunitinib and novel analogs on CRC cells, providing foundation for further investigation to combat cancer metastasis.

## 1 Introduction

Colorectal cancer (CRC) is the third most prevalent cancer and the second leading cause of cancer-related deaths worldwide. With an aging population, predictions indicate a rise in new CRC cases up to 2.3 million by 2040 (Xi and Xu, 2021). Current therapeutic options for CRC include resection surgery, chemotherapy with drugs such as oxaliplatin, 5-fluorouracil and leucovorin and radiation therapy, alone or in combination (Kumar et al., 2023). Despite notable progress in prognostic and therapeutic modalities, the overall survival rate remains moderate due to the incidence of metastasis in approximately 50% of CRC patients, presenting a substantial obstacle in disease management (Siegel et al., 2020).

Metastasis, the primary cause of cancer mortality, is an intricate process that includes the detachment of cancer cells from the primary tumor, their circulation in the bloodstream or lymphatic system, and the establishment of secondary tumors in distant or nearby organs (Welch and Hurst, 2019). In case of CRC, the lungs and liver emerge as the predominant sites for metastatic spread (Chandra et al., 2021). Epithelial-mesenchymal transition (EMT) describes the conversion of epithelial cells in carcinomas, leading to increased cell mobility and invasion capabilities, resistance to chemoradiotherapy and evasion from immune surveillance mechanisms (Huang et al., 2022). In addition to multiple signaling pathways, EMT is associated with a range of factors in the tumor microenvironment, including hypoxia, chemokines and components of the extracellular matrix (Zhang et al., 2023; Tam et al., 2020).

Hypoxia, defined as oxygen pressure below 5–10 mmHg, is a common characteristic of solid tumors resulting from insufficient vascular function and the rapid proliferation of cancer cells. Hypoxia-inducible factor-1 (HIF-1) is a heterodimeric protein comprising oxygen-regulated HIF-1α and constitutively present HIF-1β subunits. It acts as a master regulator of hypoxia-inducible genes, including transcription factors (TFs) associated with EMT like SNAIL, ZEB and TWIST families, along with cytoskeletal proteins and matrix metalloproteinases (MMPs) (Shi et al., 2021; Hapke and Haake, 2020). The stability and consequent activity of HIF-1α in hypoxic condition is closely linked to increased cell proliferation/survival, metabolic adaptability and enhanced metastatic behavior of carcinoma cells, making it a prime target for anticancer therapies.

Receptor tyrosine kinases (RTKs) play pivotal roles in angiogenesis and cell metastasis due to their strong affinity for growth factors, hormones and cytokines (Metibemu et al., 2019). Sunitinib, also known as Sutent, act as an RTK inhibitor by strongly binding to the ATP-binding pocket to obstruct kinase function. This small molecule is an FDA-approved drug for metastatic renal cell carcinoma (Motzer et al., 2017). Additionally, anticancer effects of sunitinib have been reported on medulloblastoma, meningioma, pancreas, lung and breast carcinomas (Korashy et al., 2017; Abouantoun et al., 2011; Andrae et al., 2012; Papaetis et al., 2007; Cuneo et al., 2008; Faivre et al., 2007). Sunitinib has 2,4-dimethylpyrrole moiety, and we have recently reported the synthesis of chalcone-based hydroxamic acids possessing a central 2, 4-dimethy pyrrole linker as novel analogs of sunitinib with anticancer potential (Yousefian et al., 2024).

The persistent challenge of metastasis significantly impacts the survival rates of CRC patients, underscoring the urgent need for innovative strategies to address metastatic progression. While the anticancer activity of sunitinib has been documented, there is a notable gap in research regarding its specific effect on the metastatic features of CRC cells. Accordingly, this study aimed to evaluate the effects of sunitinib on the metastatic behavior of human CRC cells and explore the potential enhancements through structural modifications. To do so, pathogenic targets of metastatic colon cancer were identified, protein-protein interaction (PPI) network was constructed, and Kyoto Encyclopedia of Genes and Genomes (KEGG) pathway enrichment analysis was conducted. Subsequently, molecular docking and dynamics simulations were carried out to define the interaction of sunitinib with HIF-1α as one of the core metastatic mediators. The expression of *HIF1A* was evaluated in seven CRC cell lines by Gene Expression Omnibus (GEO). These preliminary steps set the foundation for *in vitro* studies to examine the effects of sunitinib and novel analogs on the viability, migration, invasion and MMPs activity of CRC cells in hypoxic condition.

## 2 Methods

### 2.1 In silico analyses

#### 2.1.1 Target prediction, PPI construction and enrichment analysis

To compile a list of target proteins associated with metastatic CRC, a systematic bioinformatics approach was employed. Initially, pathogenic molecules linked to metastatic colon cancer were identified using GeneCards (https://www.genecards.org), an integrative database that provides extensive genomic, proteomic and transcriptomic information on human genes and their associated diseases. Following gene identification, PPI network was constructed using STRING database (https://string-db.org), which complies known and predicted interactions among proteins based on various types of evidence, including experimental data, computational predictions and curated databases. The resulting network was then visualized with Cytoscape software (version 3.10.1), enabling detailed analysis of complex interactions. To identify key genes within the network, CytoHubba plugin was utilized, applying the degree scoring method to pinpoint the top 40 hub genes, followed by a selection of the top 20 genes that serve as critical connectors. Finally, KEGG enrichment analysis was conducted on the identified 20 hub genes using ShinyGo online tool (version 4.1.2). This analysis assessed the biological pathways significantly associated with the hub genes, providing insights into their functional roles in metastatic CRC.

#### 2.1.2 Molecular docking

Upon defining *HIF1A* as a hub gene in metastatic colon cancer, the potential binding interactions between HIF-1α and sunitinib and analogs were defined using Autodock Vina software. The crystal structure of HIF-1α (PDB ID: 4H6J) was obtained from PDB Bank (http://www.rcsb.org/pdb) and extracted using PyMOL software. The 3D structure of sunitinib (CID: 5329102) was downloaded from PubChem (https://pubchem.ncbi.nlm.nih.gov/). The 2D structures of novel analogs were created using ChemDraw and ligand preparation was performed using Avogadro by energy minimization. AutoDockTools (version 1.5.7) was utilized to add polar hydrogens and assign atomic charges, and the output models with the most favorable energy profile were selected and visualized using Discovery Studio.

#### 2.1.3 Molecular dynamics simulations

The conformational flexibility and binding stability of the HIF-1α-sunitinib complex were investigated using GROMACS (version 2024.1). The force field parameters were derived from CHARMM General Force Field (version 4.1.65). A custom bash script was employed to merge, solvate, minimize and equilibrate the protein and ligand topologies. The TIP3P water model was used to solvate the protein-ligand complex. Subsequent neutralization was achieved by adding Cl^−^/Na^+^ ions until the maximum force per atom was below 10.0 kJ/mol. To equilibrate the system, the NVT and NPT ensembles were generated under constant temperature, volume and pressure of 300 K and 1 bar, respectively. Production runs of 100 ns were conducted with a time step of 2 fs using the leapfrog algorithm. Trajectory snapshots generated during simulations were analyzed using tools from GROMACS analysis toolkit.

#### 2.1.4 Evaluating the expression of *HIF1A* in CRC cell lines

To evaluate the expression of *HIF1A* in CRC cell lines, GEO database (https://www.ncbi.nlm.nih.gov/geo) was utilized. The analysis of microarray data informed our selection of an appropriate cell line with elevated *HIF1A* expression for further experimentation. Specifically, GSE126053 dataset was selected to perform differential expression analysis on seven CRC cell lines, which included non-metastatic HT-29, HCT116, COLO201, SW480 and SW837 cells, as well as the metastatic LoVo and T84 cells. Microarray data were obtained from GPL6848 and GPL6480 (Agilent) platforms. Data were analyzed in R (version 4.4.0) using GEOquery and limma packages. The volcano plots were visualized using ggplot2 package with *p-*value <0.05 and log_2_ fold change (log_2_FC) threshold of 0.58.

### 2.2 *In vitro* studies

#### 2.2.1 Synthesis of analogs

**FIGURE 1:**
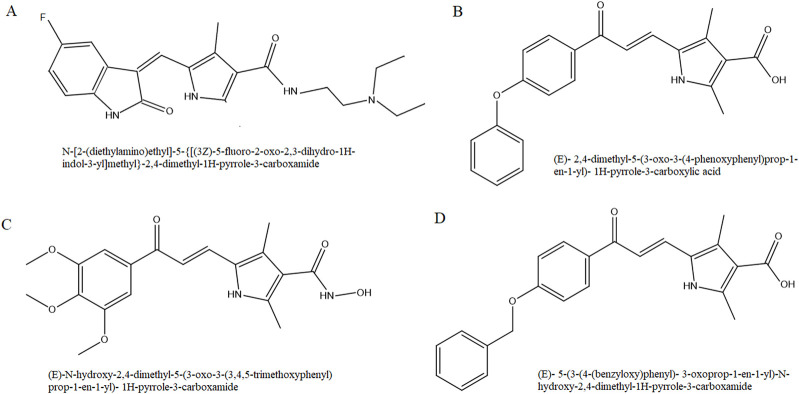
Chemical structure of sunitinib **(A)**, analog 1 **(B)**, analog 2 **(C)** and analog 3 **(D)**.

On the basis of molecular scaffold of sunitinib, we synthesized three novel analogs with chalcone-based hydroxamic acids possessing a central 2, 4-dimethypyrrole linker as recently reported (Yousefian et al., 2024). Briefly, the synthesis of 2,4-dimethyl-1H-pyrrole-3- carboxylate chalcone derivatives (analogs 1 and 3) was carried out via a reaction between 5-formyl-2,4-dimethyl-1H-pyrrole-3-carboxylic acid (Sigma) and acetophenone derivatives in methanol (Merck). Following overnight stirring of the mixture at 30°C, it was cooled and acidified, and the precipitate was recrystallized in ethanol to yield pure analog 1, (E)-2,4-dimethyl-5-(3-oxo-3-(4-phenoxyphenyl) prop-1-en-1-yl)- 1H-pyrrole-3-carboxylic acid, and analog 3, (E)-5-(3-(4-(benzyloxy)phenyl)- 3-oxoprop-1-en-1-yl)- 2,4- dimethyl-1H-pyrrole-3-carboxylic acid (Figure 1).

For the synthesis of 2,4-dimethyl-1H-pyrrole-3-carboxamide derivative (analog 2), N, N′-carbonyldiimidazole (Sigma) was added to carboxylic acid in tetrahydrofuran (Merck) and stirred. Following the addition of hydroxylamine hydrochloride (Merck), the mixture was diluted with potassium bisulfate and the organic phase was extracted with ethyl acetate. The final analog 2, (E)-N-hydroxy-2,4-dimethyl-5-(3-oxo-3-(3,4,5-trimethoxyphenyl)prop-1-en-1-yl)-1H-pyrrole-3- carboxamide, was washed with petroleum ether.

To monitor the reaction progress and preliminarily assess the homogeneity of the analogs, thin layer chromatography was performed using 250 mm Silica Gel GF Uniplates (Whatman) and dimethyl sulfoxide (DMSO, Merck) and chloroform were used as solvents. Pure analogs were visualized under ultraviolet at 254 and 365 nm. Infrared spectra (Perkin Elmer 1420 spectrometer) and ^1^H and ^13^C nuclear magnetic resonance (Bruker FT-300 MHz) were acquired to confirm all structures.

#### 2.2.2 Treatment of cells and viability assay

All *in vitro* analyses were carried out on LoVo cells, a cell line originated from a metastatic nodule resected from a patient with colon adenocarcinoma (Drewinko et al., 1976). Roswell Park Memorial Institute 1640 medium (Capricorn) supplemented with 10% fetal bovine serum (Gibco) was used for cell cultivation.

Stock solutions of sunitinib (50.18 mM), analog 1 (55.34 mM), analog 2 (53.41 mM) and analog 3 (53.33 mM) were prepared using DMSO as solvent, and final concentrations (25, 50 and 100 µM) were prepared using medium immediately before each experiment. Cells were seeded and treated in 96-well plates and incubated for 24 and 48 h in a CO_2_ incubator (Memmert) to provide 21% O_2_ (normoxic condition). For hypoxic condition, LoVo cells were seeded and treated in a similar manner but placed in a triple incubator (Binder) with a gas mixture consisting of 93% N_2_, 5% CO_2_, and 2% O_2_. To note, cells treated with an equivalent volume of DMSO (0.4% v/v) served as the solvent control in all experiments.

At the end of each incubation time, alamarBlue solution (0.1 mg/mL, Sigma) was added to cells and they were incubated in the dark for 3 h. Subsequently, absorbance was measured at 600 nm (Epoch) and cell viability (%) was calculated by the following formula; 100 – [(AT-AU/AB-AU) × 100], in which AT, AU and AB were the absorbance of treated cells, untreated cells and blank control, respectively.

#### 2.2.3 Wound healing assay

To evaluate the migration of LoVo cells upon treatment with sunitinib and analogs, wound healing assay was performed. Initially, cells (3 × 10^5^ cell/well) were seeded in 24-well plates and incubated overnight. Once a cell monolayer had formed, a vertical scratch was created using a sterile tip and wells were delicately washed with phosphate-buffered saline (PBS) to eliminate any cellular debris. Subsequently, cells were treated with 25 µM sunitinib and analogs, all prepared by serum-free medium. To assess the wound-healing capability of the cells, four microscopic fields were selected and photomicrographs were taken at 0, 24 and 48 h incubation in normoxic and hypoxic conditions. The scratch area (A) was then quantitatively analyzed using ImageJ software, and cell migration (%) was calculated by the following formula: [(A0-At)/A0] × 100.

#### 2.2.4 Boyden chamber assay

To explore the effect of sunitinib and analogs on the invasion of LoVo cells, polycarbonate members with 8 μm pores (SPL) were utilized. At first, inserts were coated with 0.1% gelatin and incubated at 37°C for 24 h. Subssequently, 15 × 10^4^ cells were treated with 25 µM sunitinib, analog 1 and analog 3 (prepared by serum-free medium) and loaded on filters, while complete medium was added to the lower chamber. The cells were then incubated in hypoxic condition for 24 h. Afterwards, filters were gently rinsed with PBS, fixed with 4% paraformaldehyde (Merck) and permeabilized with methanol. Upon staining with 10% Giemsa, photomicrographs were taken and invaded cells on the lower side of the membrane were quantified.

#### 2.2.5 Gelatin zymography

To determine whether sunitinib and analogs reduced the migration and invasion of LoVo cells via affecting MMP-2 and MMP-9 activity, gelatin zymography was performed. Briefly, cells (5 × 10^5^ cell/well) were seeded in 24-well plates and treated with 25 µM sunitinib, analog 1 and analog 3 (prepared by serum-free medium) and incubated in hypoxic condition for 24 h. The cell supernatant, containing secreted MMPs, was then collected, centrifuged at 12,000 rpm for 10 min, and mixed with loading dye composed of 25% Tris-HCl, 1% SDS, 0.01% bromophenol blue (Sigma), and 20% glycerol. Samples were then loaded into 7.5% acrylamide gel containing gelatin and electrophoresis was conducted at 150 V for 3 h. Subsequently, the separating gel was rinsed with 2.5% triton X-100 to renature MMPs, and incubated with the developing buffer, containing Tris-HCl and zinc chloride and calcium chloride, at 37°C for 18 h. Upon staining with Coomassie Brilliant blue R-250 (Merck), destaining was performed using methanol-glacial acetic acid solution for 2 h. Finally, the gels were scanned by densitometer (Bio-Rad GS-800) and ImageJ software was utilized to quantitively analyze the density of transparent bands.

#### 2.2.6 Quantitative PCR

Changes induced on the expression of EMT-TFs in LoVo cells was investigated by qPCR. Initially, cells were seeded and treated with 25 µM sunitinib, analog 1 and analog 3 in hypoxic condition and total RNA was extracted according to the manufacturer’s instruction (DENAzist Asia). cDNA was subsequently synthesized using M-MuLV reverse transcriptase (Parstous) followed by 1% agarose gel electrophoresis. qPCR was performed using SYBR green PCR master mix (Ampliqon) in PCR detection system (Bio-Rad CFX96). TBP transcripts were chosen as internal control, and normalized values were plotted as relative fold change over untreated cells. PCR cycling conditions were as follows: 94°C for 5 min, (94°C for 20 s, 58°C for 30 s, 72°C for 30 s; 34 cycles) for *TBP* (F: ACA​ACA​GCC​TGC​CAC​CTT​A and R: GAA​TAG​GCT​GTG​GGG​TCA​GT), *SNAI1* (F: GCT​GCA​GGA​CTC​TAA​TCC​AGA​GTT and R: GAC​AGA​GTC​CCA​GAT​GAG​CAT​TG) and *ZEB1* (F: CGC​AGT​CTG​GGT​GTA​ATC​GTA​A and R: GAC​TGC​CTG​GTG​ATG​CTG​AAA) primers.

#### 2.2.7 Statistical analyses

Statistical analyses were performed by One-Way ANOVA and Tukey tests using GraphPad Prism software (version 9.4.1). All experiments were carried out in triplicate for at least 3 times, and quantitative results are expressed as mean ± SD. Significant differences were defined as *p* values less than 0.05, 0.01, 0.001 and 0.0001.

## 3 Results

### 3.1 Screening of candidate targets, PPI network and KEGG enrichment analysis of hub genes

**FIGURE 2:**
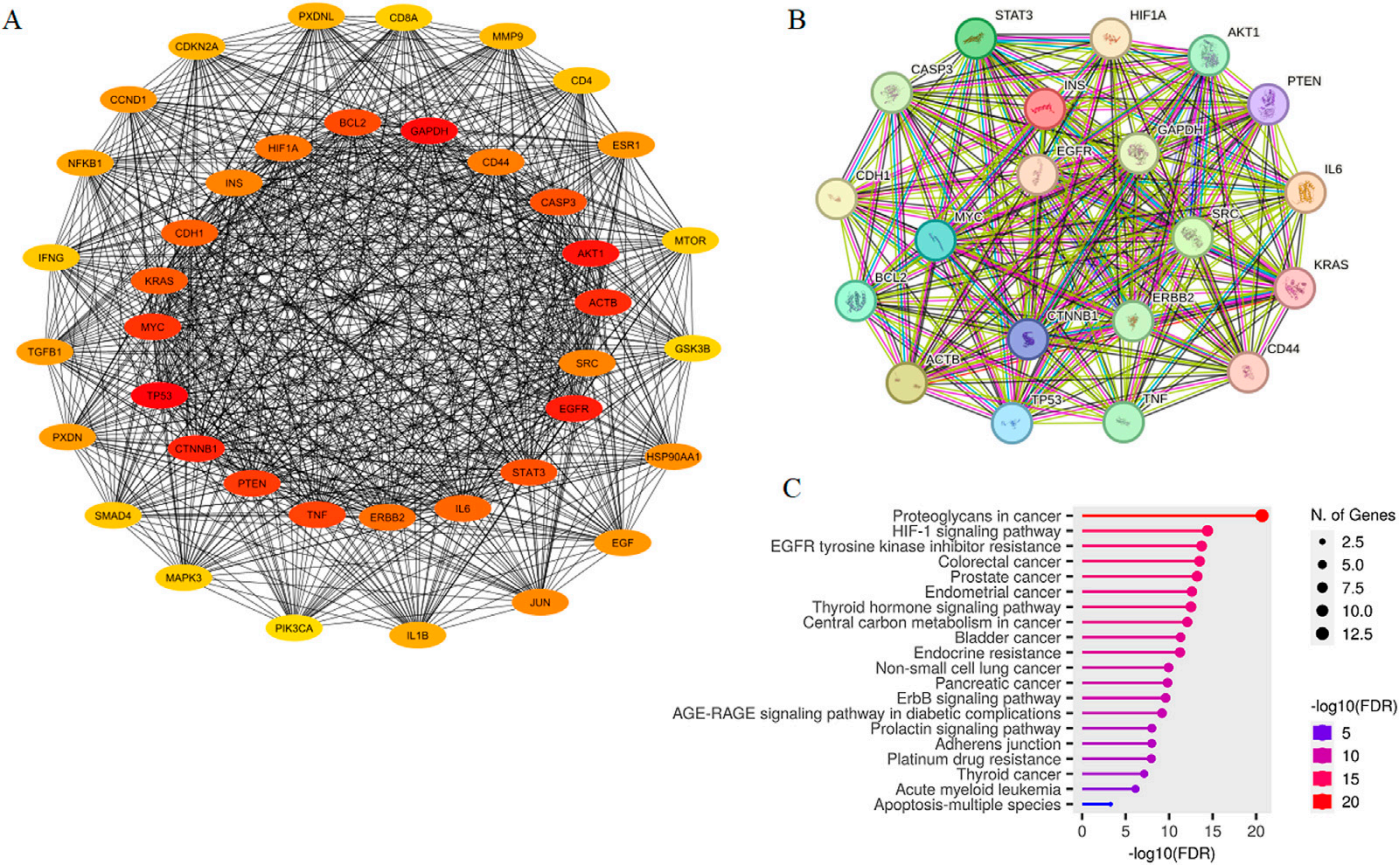
PPI Network of top 40 potential targets associated with metastatic colon cancer constructed by Cytoscape **(A)**. The network consisted of 40 nodes and 772 edges, with the top 20 hub genes located at the center, including HIF1A. The inter-connection of top 20 hub proteins was mapped using STRING **(B)**. Connections between nodes are color-coded based on the type of interaction; light blue, from curated databases; fuchsia, experimentally determined; dark green, gene-neighborhood; red, gene-fusions; dark blue, gene co-occurrence; light green, textmining; black, co-expression; purple, protein homology. KEGG pathway analysis of top 20 hub genes visualized in ShinyGO **(C)**.

Through screening targets from GeneCards database, a total of 425 potential targets associated with metastatic colon cancer were identified. These targets were then analyzed using STRING database, and the resulting network was visualized in Cytoscape. This network included 401 nodes connected by 7,465 edges (data not shown). As illustrated in Figure 2A, we identified the top 40 hub genes using CytoHubba tool, resulting in a network comprising 772 edges. Among these, the top 20 hub genes were identified, with HIF1A ranking as 17. The network of cellular regulatory proteins is depicted in Figure 2B, which includes both direct (physical) and indirect (functional) interactions among the proteins.

KEGG pathway enrichment analysis of the 20 hub genes was conducted and the results yielded *p* values (Figure 2C). Interestingly, hub genes exhibited significant enrichment in pathways “Proteoglycans in cancer”, “HIF-1 signaling pathway”, “EGFR tyrosine kinase inhibitor resistance” and “Colorectal cancer”. These pathways were found to be particularly relevant based on the gene count and statistical significance when sorted by fold enrichment. Table 1 presents KEGG enrichment results sorted by the average ranks of false discovery rate (FDR) and fold enrichment. As shown, “EGFR tyrosine kinase inhibitor resistance” (*p* = 1.8E-14), “HIF-1 signaling pathway” (*p* = 3.8E-15) and “Colorectal cancer” (*p* = 3.2E-14) were ranked as the three top pathways.

**TABLE 1:** The top ten significant KEGG pathways sorted by average ranks of FDR and fold enrichment based on *p* value*.

Description	Enrichment FDR (*p* value)	Count	Fold enrichment	Genes
EGFR tyrosine kinase inhibitor resistance	1.8E-14	8	128.7	*EGFR ERBB2 AKT1 IL6 KRAS BCL2 SRC STAT3*
HIF-1 signaling pathway	3.8E-15	9	105	*EGFR ERBB2 AKT1 GAPDH HIF1A IL6 INS BCL2 STAT3*
Colorectal cancer	3.2E-14	8	118.2	*CTNNB1 EGFR AKT1 KRAS MYC BCL2 TP53 CASP3*
Endometrial cancer	2.4E-13	7	153.4	*CTNNB1 EGFR ERBB2 AKT1 KRAS MYC TP53*
Bladder cancer	4.7E-12	6	186	*EGFR ERBB2 KRAS MYC SRC TP53*
Proteoglycans in cancer	2.0E-21	13	81.8	*CTNNB1 EGFR ERBB2 AKT1 HIF1A KRAS MYC ACTB SRC STAT3 TP53 CASP3 CD44*
Central carbon metabolism in cancer	8.1E-13	7	127.1	*EGFR ERBB2 AKT1 HIF1A KRAS MYC TP53*
Prostate cancer	6.0E-14	8	104.8	*CTNNB1 EGFR ERBB2 AKT1 INS KRAS BCL2 TP53*
Thyroid hormone signaling pathway	3.1E-13	8	84	*CTNNB1 AKT1 HIF1A KRAS MYC ACTB SRC TP53*
Endocrine resistance	5.6E-12	7	93.7	*EGFR ERBB2 AKT1 KRAS BCL2 SRC TP53*

### 3.2 Molecular docking and dynamics simulations

**FIGURE 3:**
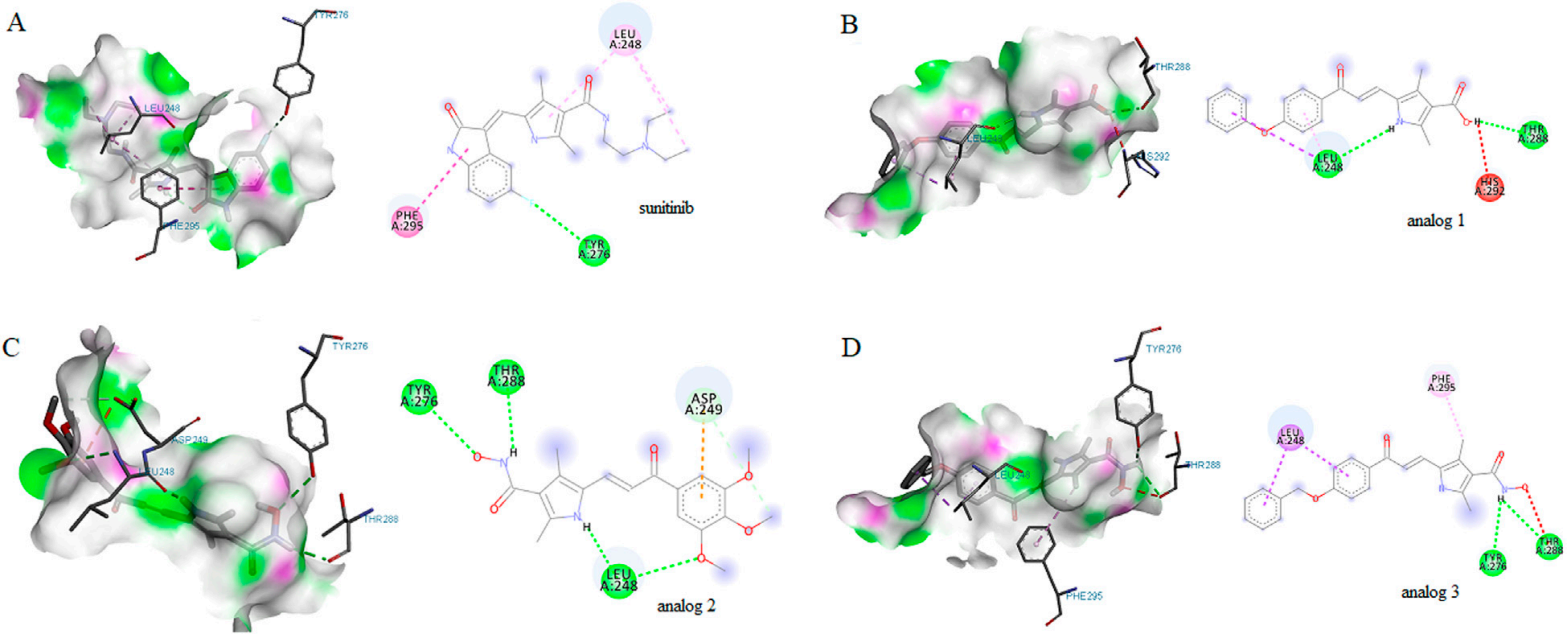
Molecular docking diagrams of sunitinib **(A)**, analog 1 **(B)**, analog 2 **(C)** and analog 3 **(D)** binding to HIF-1α PAS-B domain. 2D and 3D images were generated with Discovery Studio.

Computational analyses highlighted the importance of HIF-1α in the constructed PPI network. To investigate whether sunitinib and analogs interact with HIF-1α, molecular docking was conducted. As shown in Figure 3, results indicated the interaction of sunitinib with HIF-1α PAS-B domain, with a favorable binding affinity score of −5.4 kcal/mol. The interaction involved a hydrogen bond with specific residue at Tyr276 and van der Waals bonds at Phe295 and Leu248. The interaction between analog 1 and the residues at Thr288, Leu248 and His292 displayed a binding energy of −5.8 kcal/mol. Similarly, the interaction between analog 2 and the residues at Thr288, Leu248, Tyr276 and Asp249 displayed a binding energy of −5.2 kcal/mol. The binding positions of analog 3 and HIF-1α exhibited a binding affinity score of −6 kcal/mol, involving the formation of hydrogen bonds with residues at Tyr276 and Tyr288 and van der Waals bonds at Leu248 and Phe295.

**FIGURE 4:**
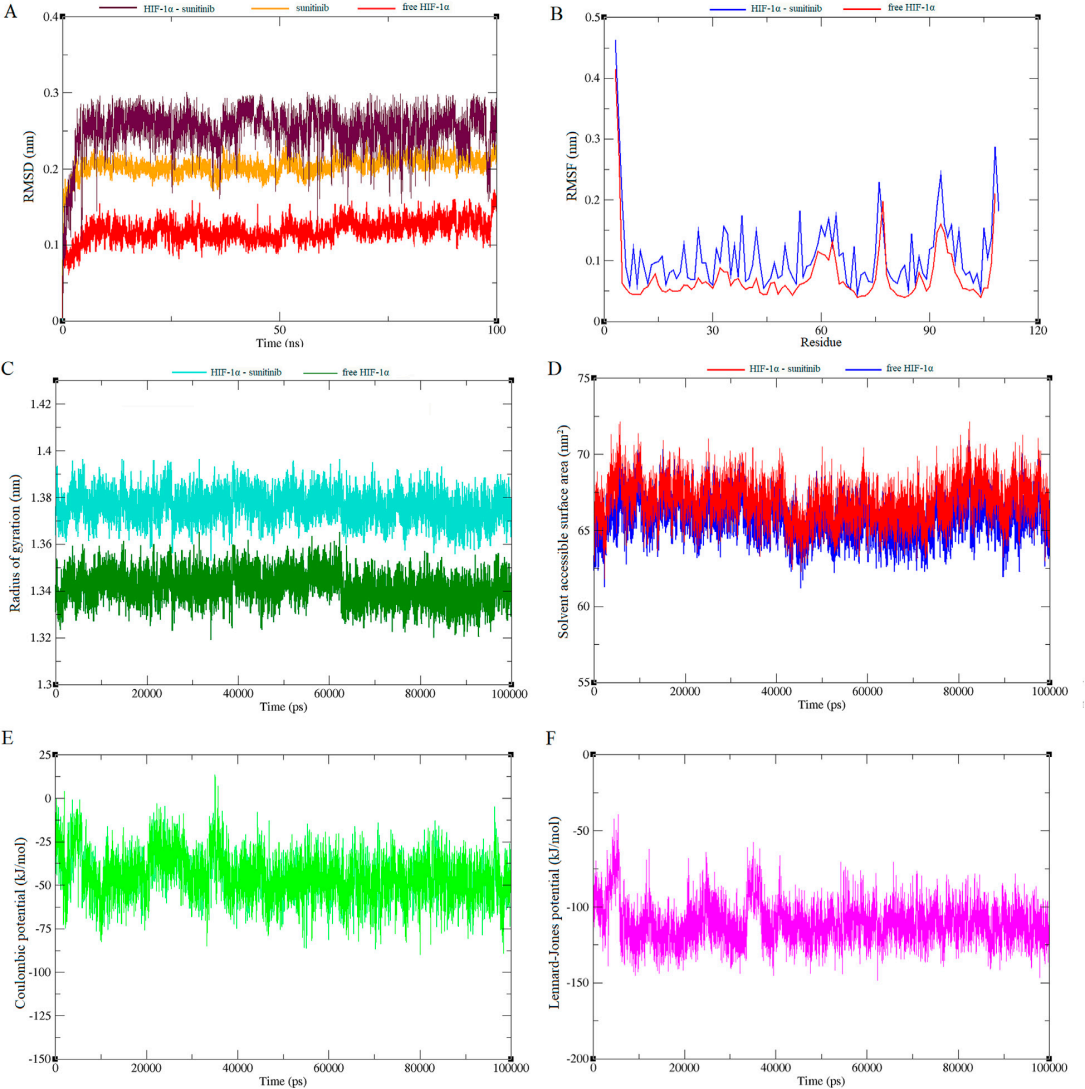
Plots generated from 100 ns molecular dynamics simulations, illustrating various structural and energetic properties of the sunitinib-HIF-1α complex. RMSD plot shows the stability of the complex over time **(A)**. RMSF plot indicates the flexibility of individual residues throughout the simulation **(B)**. Rg plot reflects the compactness of the molecular structure **(C)**. SASA plot demonstrates changes in surface exposure to solvent during the simulation **(D)**. Short-range Coulombic potential plot represents electrostatic interactions within the system **(E)**. Short-range Lennard-Jones potential plot depicts van der Waals interactions **(F)**.

Molecular dynamics simulations were also conducted on both HIF-1α and the HIF-1α-sunitinib complex to investigate the dynamic behavior of their interaction. Root mean square deviation (RMSD) graph from the simulation was examined to track the trajectories of amino acids. As depicted in Figure 4A, there were no significant changes in Cα atoms compared to the initial structure throughout the analysis. Notably, sunitinib, HIF-1α and the HIF-1α-sunitinib complex exhibited deviations below 0.3 nm. This stability in trajectories indicates that the simulation remained stable throughout the entire duration (100 ns). To further assess protein stability and investigate the impact of sunitinib on protein flexibility, mean per-residue fluctuation of the protein backbone was analyzed through root mean square fluctuation (RMSF) analysis (Figure 4B). RMSF values of all Cα atoms of HIF-1α and the HIF-1α-sunitinib complex were predominantly less than 0.4 nm, suggesting their stability. The radius of gyration (Rg), which represents the mass-weighted root-mean-square distance of atoms from their center of mass, was also analyzed to assess the stability (Figure 4C). During the simulation, both HIF-1α and the HIF-1α-sunitinib complex exhibited a consistent pattern in Rg values, falling below 1.4 nm, indicating similar levels of compactness and stability across these structures. To analyze changes in the hydrophilic and hydrophobic residues, solvent accessible surface area (SASA) was examined and results revealed that HIF-1α-sunitinib complex showed a higher SASA value than unligated HIF-1α, retaining accessibility throughout the simulation time (Figure 4D). To quantify the strength of the electrostatic and nonbonding interactions between sunitinib and HIF-1α, short-range Coulombic potential and Lennard-Jones potential were examined. The analysis indicated that sunitinib interacted with HIF-1α for the whole simulation time, further indicating the stability of their interaction (Figures 4E, F).

### 3.3 *HIF1A* expression in CRC cell lines

**FIGURE 5:**
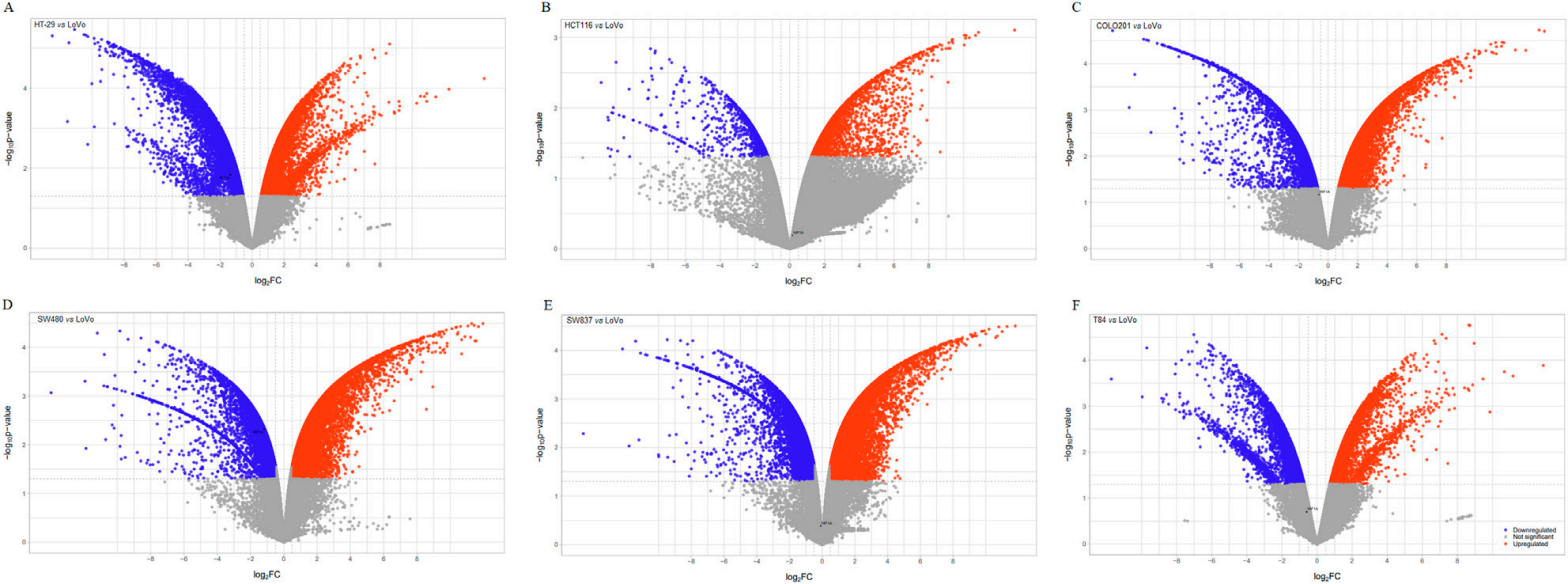
The volcano plots display differentially expressed genes in six CRC cell lines compared to LoVo cells. The *x*-axis represents the fold change (FC) in gene expression for each cell line: HT-29 **(A)**, HCT116 **(B)**, COLO201 **(C)**, SW480 **(D)**, SW837 **(E)** and T84 **(F)** relative to LoVo cells. FC threshold: 0.58, significance level: *p* < 0.05, upregulated genes: red, downregulated genes: blue.

Following the enrichment analysis that underscored the significance of HIF-1α in CRC, we evaluated *HIF1A* expression across seven CRC cell lines. The volcano plots presented in Figure 5 revealed significant downregulation of *HIF1A* in HT-29, HCT116, COLO201, SW480, SW837, and even metastatic T84 cells when compared to LoVo cells. Given that LoVo cells exhibited a higher level of *HIF1A* expression, they were deemed the most suitable choice for our experimental studies.

### 3.4 Effects of sunitinib and analogs on the viability and migration of LoVo cells

**FIGURE 6:**
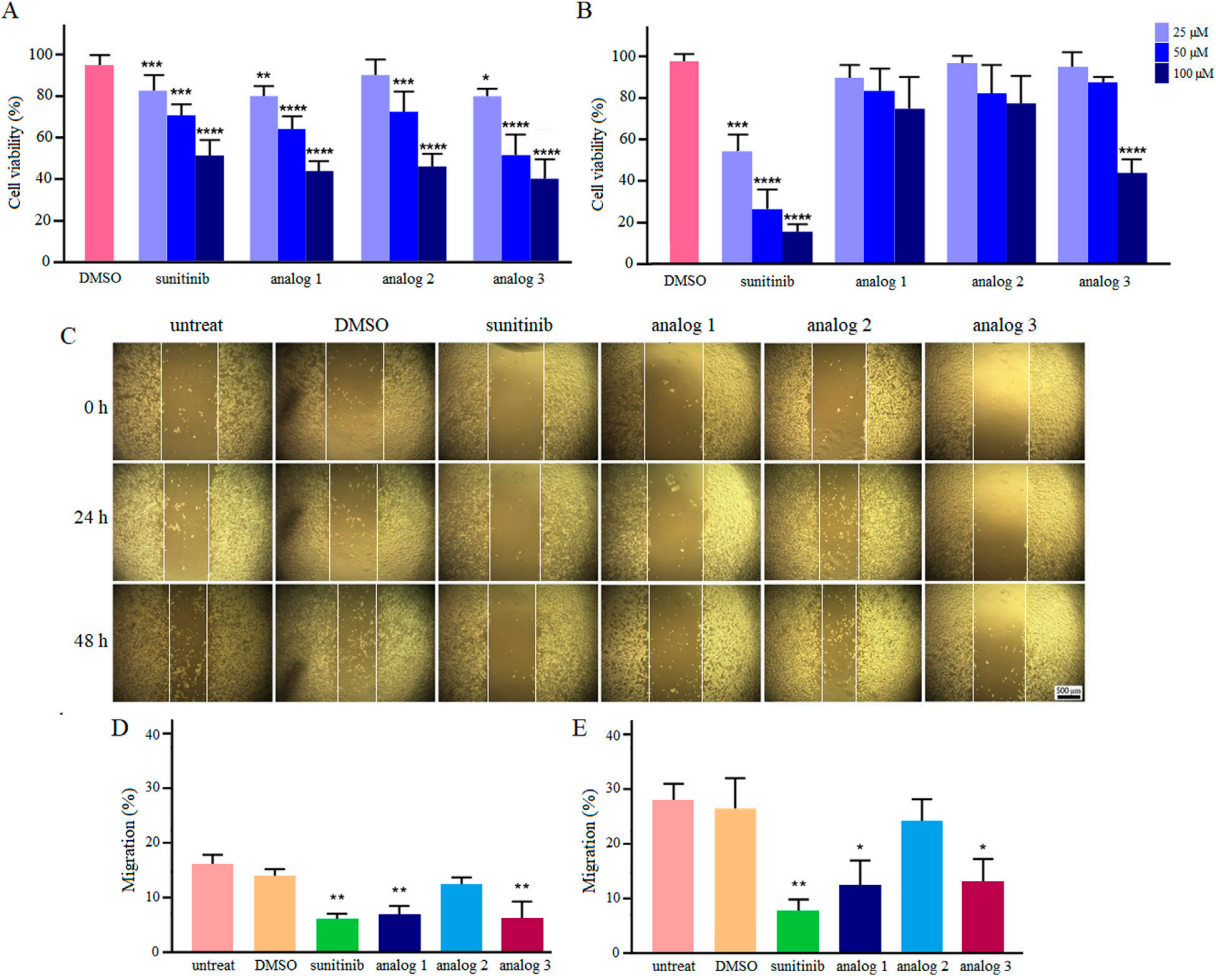
Viability and migration of LoVo cells in normoxic condition. Following treatment with sunitinib, analog 1, analog 2 and analog 3, viability was assessed and compared with the DMSO control after 24 h **(A)** and 48 h **(B)**. Photomicrographs of the scratch area during 24 and 48 h treatment with 25 μM sunitinib and analogs in comparison with untreated and 0.4% DMSO treated cells **(C)**. Quantitative analysis of LoVo cell migration after 24 h **(D)** and 48 h **(E)** compared to the DMSO control. Data are reported as mean ± SD and experiments were done for at least three times (*p < 0.05, **p < 0.01, ***p < 0.001, and ****p < 0.0001).

As predicted by computational modeling, sunitinib and analogs could interact with HIF-1α PAS-B domain and impede HIF-1α-mediated metastasis. To examine the effects of all four agents *in vitro*, it was first crucial to determine the IC_50_ values and select a sub-lethal dose. To do so, LoVo cells were treated with 25, 50 and 100 μM sunitinib and analogs and assessed for viability after 24 and 48 h. Figure 6 presents quantitative analysis of cell viability in normxic condition. Similar cytotoxicity patterns were noted across all agents after 24 h, as viability was significantly (*p* < 0.001 and *p* < 0.0001) reduced upon treatment with 50 and 100 μM sunitinib and analogs. Nevertheless, extending the treatment time up to 48 h only resulted in the improved toxicity of sunitinib, but not analogs (except 100 μM analog 3). Based on the calculated IC_50_ values (Table 2), 25 μM of each agent was chosen for wound healing assay.

**TABLE 2:** Calculated IC_50_ values of sunitinib and analogs in normoxic and hypoxic conditions.

Time (h)		IC_50_ (μM)			
		Sunitinib	Analog 1	Analog 2	Analog 3
Normoxic condition	24	106.9	84.66	91.42	65.31
	48	28.16	413.2	307.3	94.84
Hypoxic condition	24	35.36	135.7	NA	44.93
	48	48.85	241.6	NA	85.73

Figures 6C–E illustrates the effects of sunitinib and analogs on the migration of LoVo cells in normoxic condition. As shown, the migration rates of untreated and 0.4% DMSO-treated cells after 24 h were determined as 16.3% and 14.2%, respectively. However, treatment of cells with 25 μM sunitinib, analog 1 and analog 3 resulted in a substantial (*p* < 0.01) reduction in cell migration to 6.3%, 7%, and 6.3%, respectively. The migration rates calculated after 48 h were 27.5% for untreated cells and 26.1% for 0.4% DMSO-treated cells. Treatment with sunitinib, analog 1 and analog 3 significantly (*p* < 0.01, *p* < 0.05) reduced cell migration down to 7.5%, 12% and 12.7%, respectively. Analog 2 exhibited no significant effect on the migration of LoVo cells in comparison with the control groups, and the rates of migration upon 24 and 48 h treatment with this agent were determined as 12.9% and 24%, respectively. Accordingly, analog 2 was excluded from subsequent evaluations.

**FIGURE 7:**
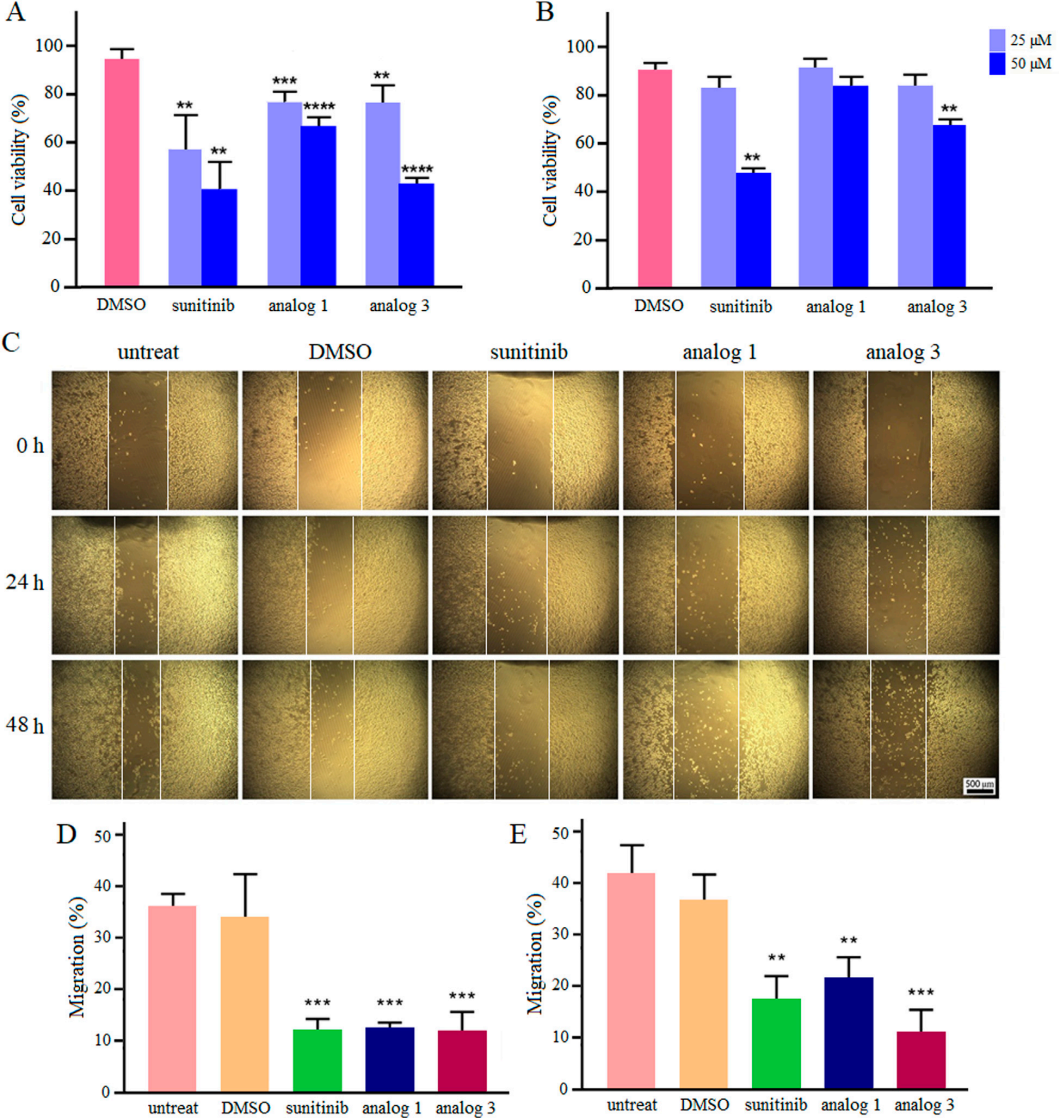
Viability and migration of LoVo cells in hypoxic condition. Upon treatment with sunitinib, analog 1 and analog 3, viability was assessed and compared with the DMSO control after 24 h **(A)** and 48 h **(B)**. Photomicrographs of the scratch area during 24 and 48 h treatment with 25 μM sunitinib, analog 1 and analog 3 in comparison with controls **(C)**. Quantitative analysis of LoVo cell migration after 24 h **(D)** and 48 h **(E)** compared to the DMSO control. Data are reported as mean ± SD and experiments were done for at least three times (*p < 0.05, **p < 0.01, ***p < 0.001, and ****p < 0.0001).

Results of viability assay in hypoxic condition are presented in Figure 7. Sunitinib, analog 1 and analog 3 induced cytotoxicity in a dose-dependent manner, with these effects being more pronounced (*p* < 0.001 and *p* < 0.0001) at 24 h compared to 48 h. Calculated IC_50_ values are presented in Table 1.

Culture of LoVo cells in hypoxic condition enhanced their migration ability. As shown in Figures 7C–E, the migration rates of untreated and 0.4% DMSO-treated cells after 24 h incubation in hypoxic condition were calculated as 35.7% and 33.9%, respectively. Interestingly, treatment with 25 μM sunitinib, analog 1 and analog 3 significantly (*p* < 0.001) reduced cell migration down to 11.9%, 12.2%, and 11.9%, respectively. Extending the incubation of untreated and 0.4% DMSO-treated cells in hypoxic condition to 48 h increased the migration rate up to 41.9% and 36.9%, respectively. Notably, treatment of cells with sunitinib, analog 1 and analog 3 resulted in substantial (*p* < 0.01 and *p* < 0.001) reduction in cell migration to 17.5%, 21.7%, and 11.3%, respectively.

### 3.5 Effects of sunitinib and analogs on the invasion, MMP activity and expression of EMT-TFs

**FIGURE 8:**
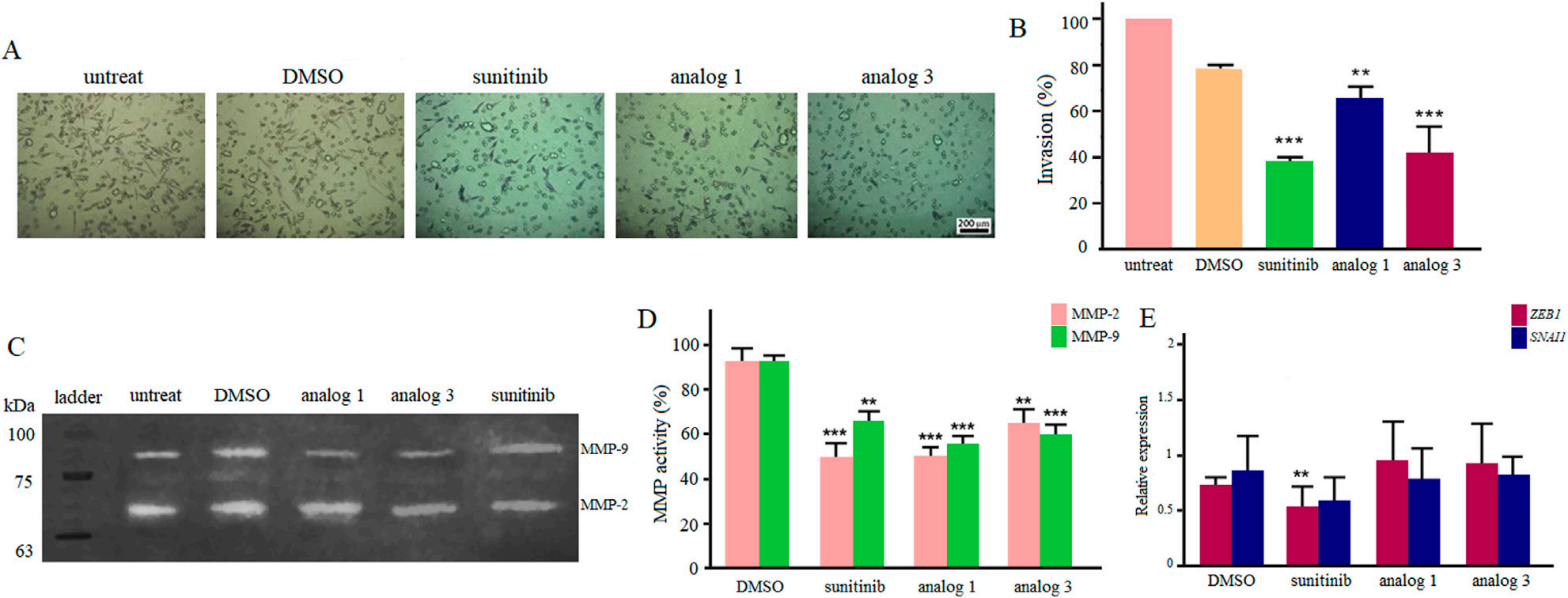
Invasion of LoVo cells upon 24 h treatment with 25 μM sunitinib, analog 1 and analog 3 in hypoxic condition **(A)**. Quantitative analysis of cell invasion between treated groups in comparison with the DMSO control **(B)**. Zymogram indicates gelatinase activity of MMP-2 and MMP-9 after 24 h treatment with 25 μM sunitinib and analogs **(C)**. Quantitative analysis of MMP-2 and MMP-9 activity compared to the DMSO control **(D)**. The expression analysis of *ZEB1* and *SNAI1* after treatment with 25 μM sunitinib and analogs compared to the DMSO control **(E)**. Data are reported as mean ± SD and experiments were carried out for at least three times (*p < 0.05, **p < 0.01, ***p < 0.001, and ****p < 0.0001).

Considering the notable inhibitory effects of sunitinib and analogs on the migration of LoVo cells in hypoxic condition at 24 h, the study further explored the invasion of cells upon the same treatment. As demonstrated in Figures 8A, B, treatment of cells with 25 μM sunitinib, analog 1 and analog 3 significantly (*p* < 0.01, *p* < 0.001) reduced the invasion of LoVo cells down to 38.2%, 64.9% and 41.4%, respectively.

Gelatin zymography was performed to assess the effects of 25 μM sunitinib and analogs on MMP-2 and MMP-9 activity, which are crucial enzymes involved in cell migration and invasion. Results presented in Figures 8C, D indicate that all agents significantly inhibited the activity of MMPs in hypoxic condition. Sunitinib and analog 1 induced more negative effects (*p* < 0.001) on MMP-2 activity than analog 3 (*p* < 0.01). On the other hand, analog 1 and analog 3 induced more substantial effects (*p* < 0.001) on MMP-9 activity rather than sunitinib (*p* < 0.01).

Results of qPCR indicated that following treatment with 25 μM sunitinib in hypoxic condition, there was a significant (*p* < 0.01) downregulation in *ZEB1* and a decrease in the expression of *SNAI1*. Regarding analog 1 and analog 3, however, no considerable alteration in gene expression was observed (Figure 8E).

## 4 Discussion

With approximately 10 million deaths attributed to cancer in 2020, the profound impact of this disease on the human health is evident. CRC is a life-threatening malignancy with an estimated substantial rise in incidence rate over the next two decades (Xi and Xu, 2021). Although conventional treatments are employed for CRC patients, the occurrence of metastasis in a significant number of cases hampers recovery rates (Sánchez-Gundín et al., 2018). Hence, the discovery of novel agents with strong anti-metastatic properties is a crucial requirement to enhance the effectiveness of therapeutic interventions. In the current study, we explored the potential of sunitinib and three novel analogs in reducing the metastatic behavior of human CRC cells for the first time.

EMT, during which epithelial cells acquire mesenchymal properties like motility and invasiveness, is associated with CRC progression and metastasis. This complex process is tightly regulated by components of the tumor microenvironment (Huang et al., 2022). Hypoxia is a common feature in most solid tumors that triggers EMT via HIFs, bHLH heterodimers composed of an oxygen-sensitive HIF-1α subunit and a constitutively expressed HIF-1β subunit. In normoxic condition, HIF-1α is continually degraded through the von Hippel Lindau-dependent ubiquitin-proteasome pathway. In hypoxic condition, however, HIF-1α is stabilized and able to translocate to the nucleus, heterodimerize with HIF-1β and activate the transcription of EMT-associated genes (Tam et al., 2020). Per-Arnt-Sim (PAS) domains (PAS-A and PAS-B) are evolutionarily-conserved regions at the N-terminus of HIF-1α and HIF-1β essential for their dimerization. The internal cavity in HIF-1α PAS-B domain serves as an allosteric binding pocket for HIF-1β PAS-B, which could be occupied by small molecules to prevent heterodimerization (Cardoso et al., 2012). Based on the pivotal role of HIF-1 in cancer metastasis, strategies targeting HIF-1α dimerization are identified as potential cancer therapeutics, although none have been successfully developed into clinically approved treatments yet (Bui et al., 2022).

Computational analyses in the present study, involving PPI construction and KEGG pathway enrichment analysis, highlighted the importance of HIF-1α as a crucial mediator of metastasis in CRC. Molecular docking was then employed to investigate the potential interaction between sunitinib and HIF-1α, as well as the impact of structural modifications on this interaction. Results demonstrated favorable interaction of sunitinib and three novel analogs with HIF-1α PAS-B domain. To define the stability of interactions, molecular dynamics stimulations were performed and stable and continuous interaction between HIF-1α and sunitinib was confirmed.

The only report on a small molecule interacting with HIF-1α PAS-B domain is for acriflavine, which blocked heterodimerization with HIF-1β and led to the suppression of tumor growth and vascularization (Yin et al., 2014). Molecular docking and dynamics analyses in the present study predicted that sunitinib and analogs could have favorable and stable interactions with HIF-1α PAS-B domain, and thus, inhibit biological activity of this protein.

Building on insights from computational modeling, we aimed to investigate whether sunitinib and its novel analogs could inhibit migration, invasion and MMPs activity in hypoxic condition. To conduct experiments, it was essential to select an appropriate CRC cell line characterized by metastatic behavior and elevated *HIF1A* expression. Analysis of microarray data identified LoVo cells as a suitable choice for our study. Following experiments demonstrated that sunitinib, analog 1 and analog 3 notably decreased the migration of LoVo cells in normoxic condition. Observing the increased motility of LoVo cells in hypoxic condition, we delved deeper into the potential of sunitinib, analog 1 and analog 3, and results revealed their considerable negative effects on cell migration and invasion in hypoxic condition. To elucidate the molecular mechanisms underlying observed inhibitory effects, gelatin zymography and qPCR were conducted, and findings indicated decreased activity of MMP-2 and MMP-9, along with downregulation of EMT-TFs in hypoxic condition.

Acting as an inhibitor of various tyrosine kinase receptors on the cell surface, sunitinib disrupts phosphorylation of intracellular domains, thereby exerting its anticancer, antiangiogenic and anti-metastatic effects (Papaetis and Syrigos, 2009). Few *in vitro* studies have demonstrated anti-metastatic potential of sunitinib. Treatment of breast carcinoma cells with this agent reduced migration, invasion and MMPs activity (Jin et al., 2016). Research on renal carcinoma cells revealed that sunitinib not only decreased MMP-2 and MMP-9 activity but also suppressed the expression of EMT markers (Yang et al., 2017). In a study conducted on non-invasive CRC cells (HT-29 cell line) in hypoxic condition, it has been shown that sunitinib suppressed HIF-1α transcriptional activity (Shin et al., 2010).

HIF-1α is known to play a pivotal role in regulating MMPs during hypoxia. Research has shown that stabilization of HIF-1α directly induces MMPs expression and activity in various cancer types (Ito et al., 2021; Shan et al., 2018; Zhang et al., 2018). The mechanistic link between HIF-1α and MMP regulation is further supported by evidence showing that inhibiting HIF-1α leads to downregulation of MMPs (Tsai et al., 2016). Consequently, sunitinib and its analogs may inhibit metastasis not only by directly affecting MMP activity in hypoxic condition, but also by disrupting the upstream signaling pathways involving HIF-1α.

To strengthen our findings and establish sunitinib and its analogs as promising therapeutic agents against metastatic CRC, several key research directions should be pursued. First, it is crucial to experimentally verify the binding interactions between sunitinib and analogs with HIF-1α. This will clarify whether these agents inhibit the transcriptional activity of HIF-1α directly or exert their effects through its downstream targets. Furthermore, incorporating animal models in future studies will be vital for evaluating the efficacy and safety of sunitinib and analogs in a biological context that closely resembles human disease.

## 5 Conclusion

Despite significant advancements in cancer treatment, the mortality rate attributed to cancer metastasis remains a pressing concern. The current study investigated effects of sunitinib and novel analogs on the metastatic behavior of CRC cells for the first time. Molecular docking and dynamics simulations indicated favorable and stable interactions of sunitinib and novel analogs with HIF-1α PAS-B domain, which was translated to reduced migration, invasion and MMPs activity in hypoxic condition. Our findings open up avenues for the development of new therapeutics against cancer metastasis. As this study focused on computational modeling and *in vitro* analyses, results need validation through *in vivo* studies and clinical trials to assess therapeutic efficacy and safety of these agents.

## Data Availability

The original contributions presented in the study are included in the article/supplementary material, further inquiries can be directed to the corresponding author.
